# Localization and Trafficking Behavior of SPPL2A During Mouse Spermiogenesis

**DOI:** 10.3390/cells15191773

**Published:** 2026-09-29

**Authors:** Cheng Zheng, Wei Li, Shahnaz Parveen, Anuradha Yadav, Kristina Bennett, Jie Xu, Jifeng Zhang, Ling Zhang, Christopher V. Kelly, Zhibing Zhang

**Affiliations:** 1 Department of Environmental Health and Occupational Medicine, Academy of Nutrition and Health, Hubei Province Key Laboratory of Occupational Hazard Identification and Control, School of Public Health, Wuhan University of Science and Technology, Wuhan 430065, China; soumns_1216@163.com; 2 Department of Physiology, Wayne State University, 275 E Hancock Street, Detroit, MI 48201, USA; waylee@wayne.edu (W.L.); hd9974@wayne.edu (K.B.); 3 Department of Physics and Astronomy, Wayne State University, Detroit, MI 48201, USA; parveen@wayne.edu (S.P.); anuradhayadav@wayne.edu (A.Y.); 4 Barber Center for Multiscale Systems Biology, Wayne State University, Detroit, MI 48201, USA; 5 Center for Advanced Models for Translational Sciences and Therapeutics, University of Michigan Medical School, Ann Arbor, MI 48109, USA; jiex@med.umich.edu (J.X.); jifengz@umich.edu (J.Z.); 6 Department of Obstetrics and Gynecology, Wayne State University, Detroit, MI 48201, USA

**Keywords:** SPPL2A, spermiogenesis, sperm flagellum, intraflagellar transport, single-particle tracking

## Abstract

Signal peptide peptidase-like 2A (SPPL2A) is an intramembrane protease identified as a binding partner of intraflagellar transport protein 27 (IFT27), suggesting a potential role in sperm flagellum development. However, the localization and trafficking of SPPL2A during spermiogenesis remain unknown. Here, we generated an endogenous SPPL2A–mCherry knock-in mouse model using CRISPR/Cas9 to visualize SPPL2A in living male germ cells. Expression of the fusion protein was confirmed by Western blotting, and fertility, spermatogenesis, sperm morphology, and motility were evaluated. Live-cell fluorescence imaging, immunofluorescence, and single-particle tracking (SPT) were used to characterize SPPL2A localization and trafficking. Homozygous knock-in mice exhibited normal fertility, spermatogenesis, and sperm parameters, indicating that the mCherry fusion did not disrupt SPPL2A function. SPPL2A–mCherry was detected as punctate structures in developing germ cells and elongating sperm tails. Its localization changed dynamically during spermiogenesis and was closely associated with developing tail microtubules. Live-cell SPT demonstrated bidirectional movement of SPPL2A-containing particles within developing sperm tails. Mean-squared displacement analysis indicated predominantly confined diffusion with a confinement length scale of 153 ± 15 nm, while particle trajectories showed a slight bias away from the cell body. Particle mobility progressively decreased during sperm maturation, with highly mobile puncta in early spermatids becoming largely immobile in mature sperm. These findings establish an endogenous SPPL2A fluorescent knock-in mouse model and show that SPPL2A-containing particles exhibit intracellular movement within developing sperm tails during spermiogenesis. This model provides a valuable platform for investigating protein transport during sperm flagellum assembly.

## 1. Introduction

Motile cilia and flagella are specialized organelles composed of microtubules, crucial for cellular movement, fluid dynamics, and signal transduction [1]. The sperm flagellum in male germ cells is essential for sperm motility and successful fertilization. Its creation relies on the meticulously coordinated assembly of axonemal structures, auxiliary cytoskeletal components, and membrane-associated proteins throughout spermiogenesis [2,3]. Furthermore, because mature sperm flagella are devoid of intrinsic protein synthesis capabilities, the accurate transfer of structural and regulatory proteins from the cytoplasm to the developing flagellum is fundamentally dependent on intraflagellar transport (IFT) [4].

IFT is facilitated by two multimeric protein complexes, IFT-A and IFT-B, which collaborate with kinesin-2 and dynein-2 motors to enable bidirectional transport along axonemal microtubules [4,5,6]. In somatic cilia, IFT is essential for ciliary assembly, maintenance, and signaling. In spermatogenesis, mounting evidence suggests that IFT is essential for the creation of the sperm flagellum, including axonemal elongation, fibrous sheath organization, and mitochondrial sheath assembly [7]. Moreover, genetic mutations in various IFT components, including IFT27, IFT20, IFT25, and IFT172, have been demonstrated to result in aberrant flagellar morphogenesis and male infertility in both humans and animal models, highlighting the essential function of IFT in spermiogenesis [8,9,10].

IFT27, a small Rab-like GTPase in the IFT-B complex, is crucial for regulating IFT train modification, BBSome recruitment, and the loading and turnover of membrane-associated cargoes during cilia and flagella formation [11,12]. In male germ cells, IFT27 is crucial for sperm flagella synthesis; its deficiency results in defective axonemal elongation, abnormal accessory structures, and complete male sterility [8]. Our previous yeast two-hybrid screening identified SPPL2A as a potential binding partner of IFT27. Signal peptide peptidase-like 2A (SPPL2A) is an intramembrane aspartyl protease belonging to the signal peptide peptidase-like (SPPL) family [13]. Extensive research on immune cells has demonstrated that SPPL2A regulates the maturation of B-cells and dendritic cells through the intramembrane cleavage of type II transmembrane proteins [14,15]. Nevertheless, despite its well-defined role in the immune system, the expression profile and biological significance of SPPL2A in the male reproductive system remain largely unexplored.

The relationship between SPPL2A and IFT27 indicates an undiscovered connection among intramembrane proteolysis, vesicle trafficking, and IFT-mediated flagellar assembly. The endogenous distribution, developmental dynamics, and potential trafficking behavior of SPPL2A in spermatogenic cells are entirely unknown, creating a substantial knowledge gap that hinders our comprehension of the processing and delivery of membrane-associated cargos during sperm flagellum formation.

## 2. Materials and Methods

### 2.1. Ethics Statement

All animal procedures received approval from the Institutional Animal Care and Use Committee (IACUC) at Wayne State University and were performed in accordance with the National Institutes of Health Guide for the Care and Use of Laboratory Animals. Adult mice were euthanized by CO_2_ inhalation followed by cervical dislocation as a secondary physical method. The testes and epididymides were immediately collected after euthanasia and processed according to the requirements of the corresponding experiments.

### 2.2. Generation of SPPL2A-mCherry Knock-In Mice

The SPPL2A-mCherry knock-in (KI) mouse model was generated using the CRISPR/Cas9 system at the Transgenic Animal Model Core of the University of Michigan. The mouse SPPL2A protein was fused with an mCherry tag at the C-terminus. Briefly, the mCherry coding sequence, followed by a bovine growth hormone polyadenylation (bGH PA) signal, was inserted immediately before the stop codon within exon 15 of the *Sppl2a* gene. The DNA donor was modified to prevent Cas9 re-cleavage after homologous recombination. To improve long single-stranded DNA synthesis efficiency, the poly-T repeat within the donor was shortened, and multiple silent wobble-base substitutions were introduced into the mCherry sequence to reduce GC content.

A validated single-guide RNA (sgRNA C328G1) targeting the terminal exon of *Sppl2a* was selected. Cas9 was used to introduce a double-strand break near the endogenous termination codon of *Sppl2a*. Homology-directed repair (HDR) of the chromosomal break using a long single-stranded DNA donor placed the mCherry-bGH PA cassette immediately upstream of the termination codon. The sgRNA was tested and confirmed to be active prior to in vivo microinjection.

To identify founder mice, genomic DNA was extracted, and PCR genotyping was performed using primers spanning the knock-in region. The primer pair used for screening was: Sppl2a-mCherry span Forward: 5′-GCCTCAAATGTACATCCACATATGCAAAA-3′. Sppl2a-mCherry span Reverse: 5′-TCGCTGTAAATGTTCCCAATTTTCAACTC-3′. PCR amplification generated a 1356 bp product from the knock-in allele, whereas no corresponding band was detected in wild-type (WT) controls. The founder mice carrying the correctly targeted knock-in allele were subsequently crossed with WT mice to establish the SPPL2A-mCherry KI lines (F1 generation). These F1 generation mice were crossed again with WT mice, and each line was examined by Western blot analysis to see if the fusion SPPL2A-mCherry protein was expressed.

### 2.3. Male Fertility Test

2–3-month-old KI male mice and age-matched WT controls were used for fertility assessment. Each male was housed with a sexually mature WT female (2–3 months old) in a one-to-one mating scheme for at least 4 consecutive weeks. Females were examined daily for the presence of vaginal plugs, and successful pregnancy was monitored thereafter. Once pregnancy was confirmed, females were separated into individual cages. The number of pregnant females and litter size per delivery were recorded to evaluate male fertility.

### 2.4. Spermatozoa Counting and Motility Assay

Spermatozoa were obtained from the cauda epididymides and allowed to disperse in pre-warmed phosphate-buffered saline (PBS). For sperm counting, aliquots of sperm suspensions were fixed in 4% formaldehyde for 10 min at room temperature, washed twice with PBS, and then loaded onto a hemocytometer chamber. Sperm concentration was calculated according to standard procedures.

For sperm motility analysis, freshly isolated spermatozoa were maintained under non-capacitating conditions and examined using an inverted microscope (Nikon, Tokyo, Japan). Time-lapse images were recorded at 15 frames per second, and ten randomly selected fields were analyzed for each sample. Individual sperm tracks were analyzed to calculate curvilinear velocity (VCL), defined as the curvilinear distance (DCL) traveled by a sperm cell per second (VCL = DCL/t). In parallel, sperm motility parameters were additionally assessed using an IVOS II computer-assisted sperm analysis (CASA) system (Hamilton Thorne Inc., Beverly, MA, USA).

### 2.5. Western Blot Analysis

Testicular tissues were lysed in ice-cold lysis buffer using an Ultra-Turrax tissue homogenizer, followed by brief sonication with an ultrasonic processor (Qsonica, Newtown, CT, USA) at 70% amplitude for 10 s. The lysates were kept on ice for 30 min and subsequently centrifuged at 12,000 rpm for 20 min at 4 °C. Supernatants were collected, and protein concentrations were determined using a DC protein assay kit (Bio-Rad, Hercules, CA, USA). Equal amounts of protein were denatured by heating, resolved on 12% SDS–polyacrylamide gels, and transferred onto PVDF membranes (Millipore Sigma, Burlington, MA, USA). The membranes were blocked with 5% non-fat milk at room temperature for 1 h, followed by incubation at 4 °C overnight with primary antibodies (mCherry: Cat. No. 56808-1-Ig, Proteintech, 1:1000; β-actin: Proteintech, 1:10,000). After extensive washing, membranes were incubated with appropriate HRP-conjugated secondary antibodies (goat anti-mouse IgG (H+L), Cat. No. 626520; goat anti-rabbit IgG (H+L), Cat. No. 656120; Invitrogen; both at 1:2000 dilution) for 1 h at room temperature. Chemiluminescent signals were visualized using a Pico Ultra ECL Western blot detection kit (Lambda Biotech Corporation, Ballwin, MO, USA; Cat. No. G075).

### 2.6. Histological Analysis and H&E Staining

Testes and epididymides were collected immediately after euthanasia, and the surrounding connective and adipose tissues were carefully removed. The testes were weighed before fixation. Testicular and epididymal tissues were fixed in 4% paraformaldehyde overnight at 4 °C, dehydrated through a graded ethanol series, cleared in xylene, embedded in paraffin, and sectioned at a thickness of 4 μm.

For hematoxylin and eosin staining, the paraffin sections were deparaffinized in xylene twice for 5 min each and rehydrated through 100%, 95%, and 70% ethanol to distilled water. The sections were stained with hematoxylin for 5 min, rinsed in running tap water for 5 min, differentiated in 1% acid alcohol for 5–10 s, and rinsed again in running tap water. The sections were subsequently counterstained with eosin for 2 min, dehydrated through graded ethanol, cleared in xylene twice for 5 min each, and mounted using a permanent mounting medium. Histological images were acquired using an Aperio ScanScope whole-slide imaging system (Leica Biosystems, Nussloch, Germany).

### 2.7. Fluorescence Analysis of Testicular Cells

Testes were freshly dissected from adult mice and immediately fixed in 4% paraformaldehyde in 0.1 M PBS (pH 7.4) overnight at 4 °C. For fresh germ cell isolation, testes were enzymatically digested in 0.5 mg/mL collagenase IV and 1.0 μg/mL DNase I (Sigma-Aldrich, Burlington, MA, USA) at 32 °C for 30 min with gentle stirring to release individual germ cells. Freshly isolated germ cells were immediately diluted in PBS and observed by spinning-disk confocal microscopy (Andor Dragonfly 201) at room temperature. For fixed-cell imaging, a subset of cells was fixed with 4% paraformaldehyde containing 0.1 M sucrose at room temperature prior to imaging. After dehydration through a graded ethanol series, tissues were embedded in paraffin, and 5-μm sections were prepared using a Leica paraffin microtome. Sections were dewaxed in xylene and rehydrated through a series of descending ethanol concentrations. For antigen retrieval, sections were heated in either 10 mM Tris–EDTA buffer (pH 9.0) or 10 mM sodium citrate buffer (pH 6.0) for 30 min, followed by cooling to room temperature. After rinsing in PBS, sections were blocked with 10% goat serum at room temperature for 1 h and incubated overnight at 4 °C with the appropriate primary antibodies diluted in blocking buffer. After washing with PBS, sections were incubated with corresponding Alexa Fluor–conjugated secondary antibodies for 1 h at room temperature in the dark. For acrosome staining, sections were incubated with Alexa Fluor 488–conjugated peanut agglutinin (PNA-488) (1:4000 dilution) for 1 h at room temperature after serum blocking. Nuclei were counterstained with DAPI, and sections were mounted using an anti-fade mounting medium.

In testicular sections, germ cell types were identified based on their relative positions within the seminiferous epithelium and their nuclear morphology. Spermatogonia were identified as cells located adjacent to the basement membrane with round or oval nuclei. In isolated germ cells, spermatid developmental stages were assigned according to nuclear morphology and stage-associated structures. Round spermatids were characterized by small, round nuclei and stage-dependent acrosomal morphology visualized by PNA staining. Elongating spermatids were identified by progressive nuclear elongation and chromatin condensation, together with the presence of an α-tubulin-positive manchette. Late-stage spermatids were characterized by highly condensed, hook-shaped nuclei and elongated flagella. When an exact spermatid step could not be determined from nuclear morphology alone, the developmental stage was reported as an approximate step range.

### 2.8. Fluorescence Imaging and Single-Particle Tracking Analyses

Fluorescence and brightfield images were captured using a spinning-disk confocal microscope (Dragonfly 201, Andor Technology Ltd., Belfast, Northern Ireland, UK) mounted on an inverted microscope (Leica DMI8) with a 63× oil immersion objective (HC PL APO, Leica Microsystems GmbH) and an sCMOS camera (Zyla 4.2 PLUS, Andor Technology Ltd.). Consistent acquisition settings were used for all color channels, and the images were uniformly displayed with the same brightness and contrast adjustments.

For single-particle tracking analysis, cells were rinsed with HEPES and pipetted onto a 35 mm glass-bottom plate for imaging to assess SPPL2 trafficking. Brightfield and epifluorescence imaging were performed on an inverted microscope (IX83, Evident Scientific, Inc., Tokyo, Japan) equipped with a 100×, 1.49 NA objective and a 2× magnification lens. Fluorescence illumination was performed with a free-space 15-mW, 561-nm laser (OBIS, Coherent Technology Corp., Saxonburg, PA, USA), cleaned by a bandpass filter (ZET405/488/561/647X, Chroma Technology Corp., Bellows Falls, VT, USA), reflected by a quad-band dichroic mirror (ZT405/488/561/647RPC, Chroma Technology Corp.), and distributed across a 2000 µm^2^ sample area. Emission passed through a 525/50M emission filter (Evident Scientific, Inc.) and a 4-band notch filter (ZET405/488/561/640m; Chroma Technology Corp.) before being detected by an EMCCD camera (iXon 897-Ultra, Andor Technology Ltd.) with 150 EM gain, 20 ms exposure, and 47 Hz frame rate.

ImageJ/Fiji (ImageJ 1.54p) and custom Python scripts (Python 3.11.14, conda-forge) analyzed videos > 15 s in duration to extract and fit kymographs. The linking and preliminary analysis of the single-particle tracking data were performed with TrackPy. Mean-squared displacement (MSD) versus delta time (Δt) curves were fit with a 1D two-regime model that accounted for localization uncertainty (σ), a short-time diffusion rate with minimal restrictions to motion ($D_{short}$), a long-time diffusion with motion affected by confinement of SPPL2A–mCherry to tethering molecules or similar structures ($D_{long}$), and a crossover time between these regimes ($\tau_{c}$), according to Equation (1):

(1) MSDΔt= 2DshortΔt+σ,  2DlongΔt−τc+2Dshortτc+σ, Δt≤ τcΔt> τc

### 2.9. Statistical Analyses

For this research, we utilized GraphPad Prism software (GraphPad Software, San Diego, CA, USA) to conduct the statistical analyses. The normality of the dataset was verified using the Shapiro–Wilk test, while the homogeneity of variances among groups was established through the Levene test. Provided that the quantitative data met the criteria for normal distribution and equal variances, Student’s *t*-test was applied. A result was considered statistically significant when the *p*-value was below 0.05.

## 3. Results

### 3.1. Generation of SPPL2A-mCherry KI Mice

**Figure 1:**
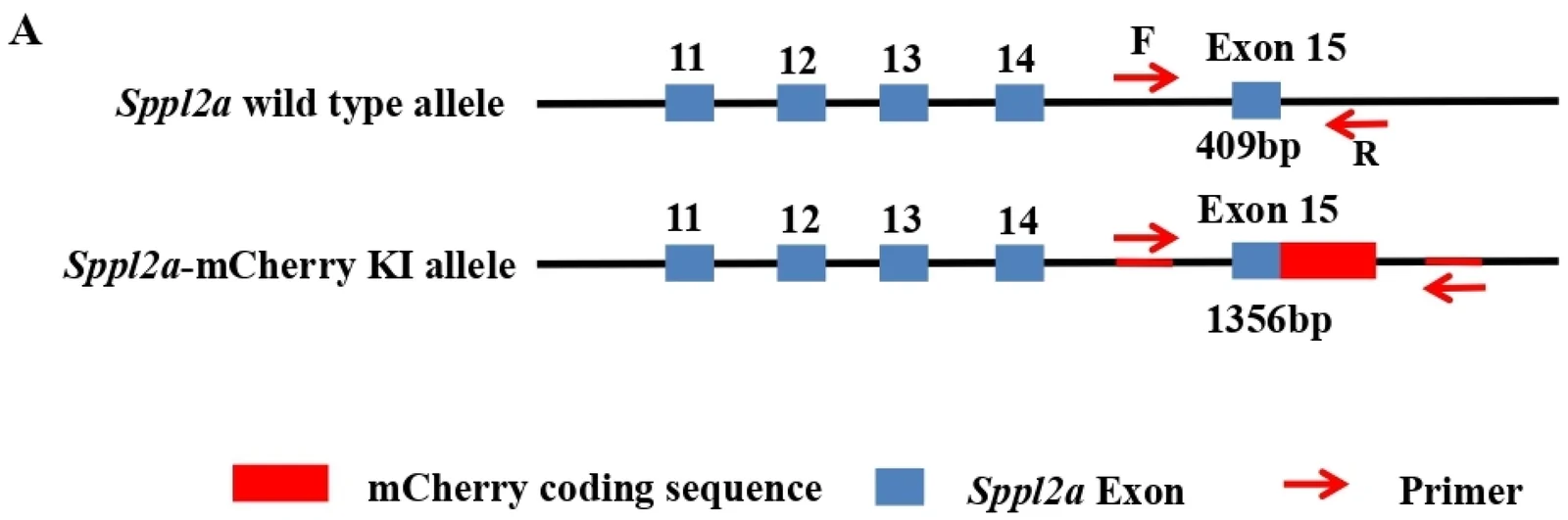

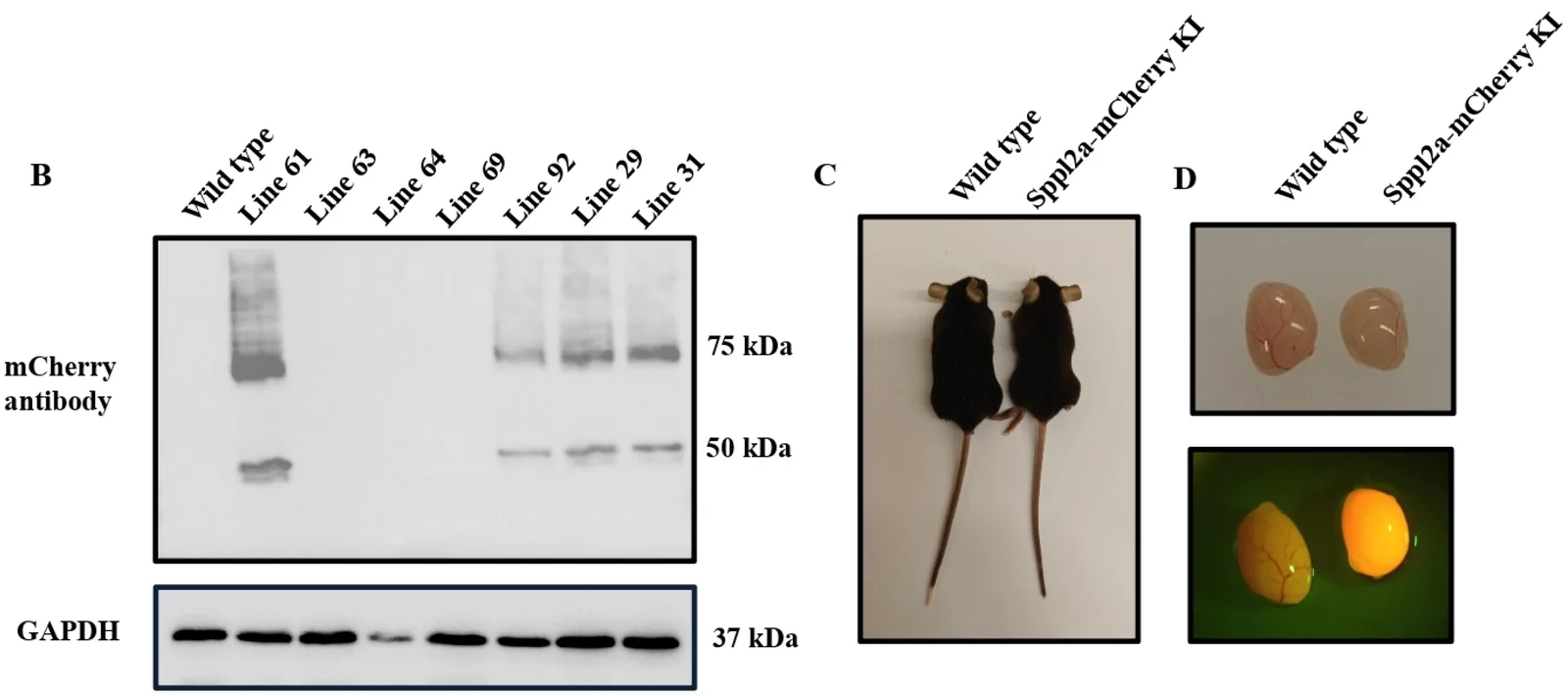
Generation of a Sppl2a–mCherry KI mouse model. (**A**). Schematic representation of the strategy used to generate the Sppl2a–mCherry KI mouse model. The mCherry coding sequence was inserted in-frame immediately before the stop codon of exon 15 of the mouse *Sppl2a* gene. The positions of the genotyping primers and the expected PCR product sizes for the wild-type (409 bp) and knock-in (1356 bp) alleles are indicated (**B**). A representative Western blot result of testicular lysates from different KI lines using an anti-mCherry antibody. GAPDH served as a loading control. Two fusion proteins with different sizes (75 kDa and 50 kDa) were present in some KI lines (positive), and these proteins were not present in the wild-type mice or other KI lines (negative). The two fusion proteins indicated that mCherry was fused to the full-length SPPL2A (Sppl2a-201) and a shorter isoform (Sppl2a-203 in the Supplemental Figure S1). (**C**). A representative gross image of a WT mouse and a Sppl2a–mCherry knock-in (KI) littermate. No visible difference was observed between WT and KI mice. (**D**). Examination of the testes. Upper: bright-field images of testes from a 3-month-old KI mouse and its WT littermate; lower: red fluorescence was specifically detected in the testes of the KI mouse under a fluorescence dissecting microscope.

The knock-in strategy employed to generate the KI mouse model is illustrated in Figure 1A and Supplementary Figure S1. Genotyping was performed using primers flanking the C-terminal region of exon 15, yielding PCR products of 409 bp for the wild-type allele and 1356 bp for the knock-in allele (Figure 1A and Supplementary Figure S2). A Western blot was conducted using testicular extracts from each line with an anti-mCherry antibody to examine expression of the SPPL2A-mCherry fusion protein. Each independent PCR-positive line was further evaluated by fluorescence imaging of freshly dissected testes and by Western blotting of testicular extracts using an anti-mCherry antibody. Lines without detectable testicular red fluorescence or SPPL2A–mCherry fusion protein expression were excluded from subsequent experiments. Only expression-positive lines were maintained for further analysis. In these lines, Western blotting detected two mCherry-positive protein bands: an approximately 75-kDa band corresponding to the predicted full-length SPPL2A–mCherry fusion protein (SPPL2A-201) and an approximately 50-kDa band that may correspond to the shorter SPPL2A-203 isoform (Figure 1B and Supplementary Figure S1A).

Homozygous KI mice exhibited normal development during routine observations, with some animals monitored for up to eight months (Figure 1C). Throughout the study, the gross morphology of the testes in both WT and KI mice remained comparable (Figure 1D, upper panel). Under fluorescence microscopy, strong red fluorescent signals were clearly visible in the testes of KI mice, whereas WT controls showed no fluorescence, confirming that mCherry had been successfully fused to the endogenous SPPL2A protein (Figure 1D, lower panel).

### 3.2. Fertility and Sperm Parameters Were Normal in the KI Mice

To evaluate the reproductive capability of the KI mice, 2-month-old homozygous KI males and age-matched WT males were each paired with 2-month-old WT females. The KI males produced litters with normal pregnancy rates and average litter sizes compared with WT controls (Table 1), indicating that mCherry insertion did not impair male fertility.

**Figure 2:**
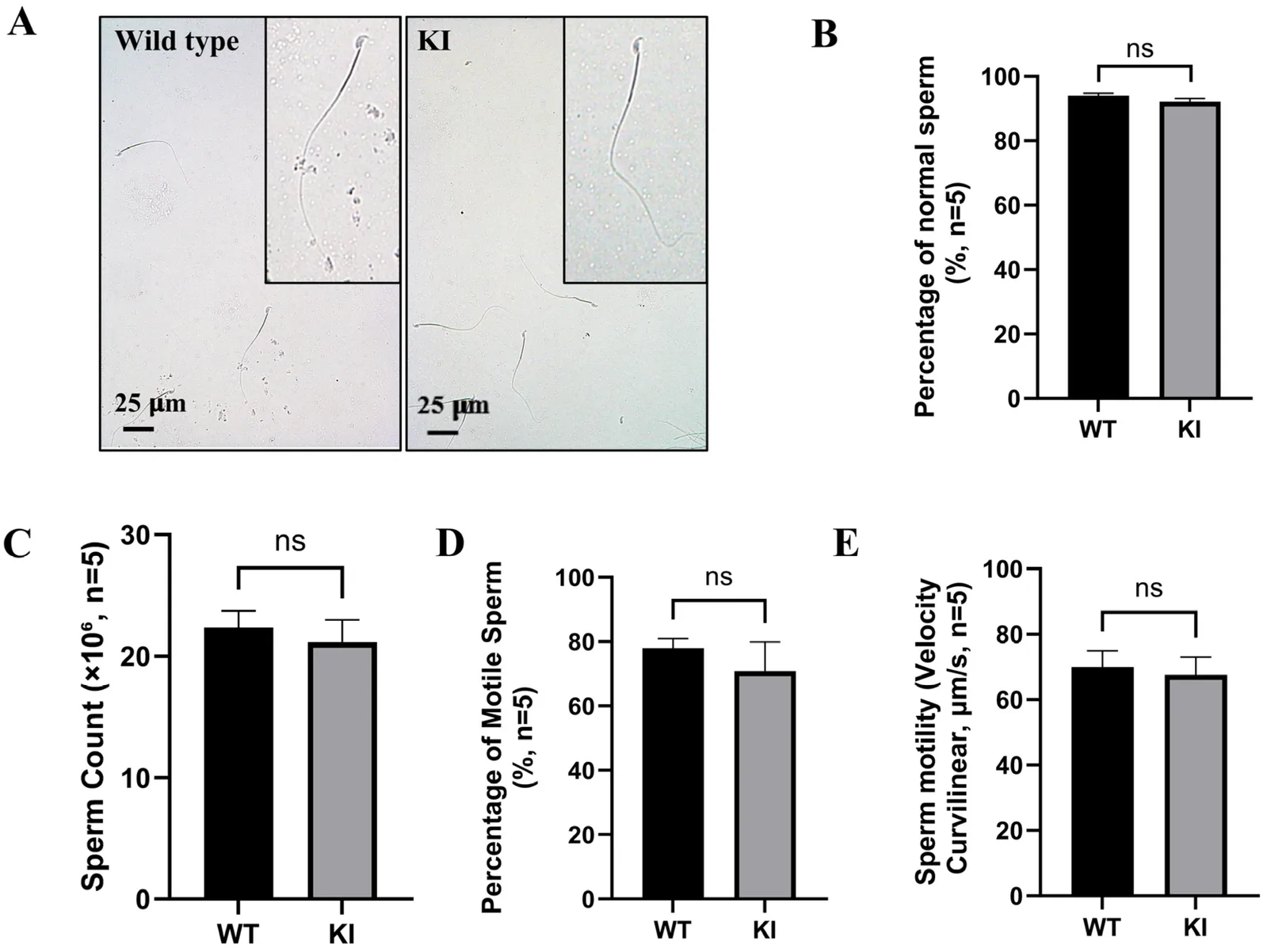
Analysis of sperm parameters in WT and Sppl2a–mCherry KI mice. (**A**). Representative bright-field images showing normal sperm morphology from WT and KI mice; (**B**). Percentage of morphologically normal sperm in WT and KI mice (%, *n* = 5); (**C**). Sperm count (10^6^, *n* = 5). (**D**). Percentage of motile sperm (%, *n* = 5); (**E**). Sperm motility assessed by curvilinear velocity (VCL, µm/s, *n* = 5). ns: No significant difference was observed between the WT mice and the KI mice.

Spermatozoa collected from the cauda epididymides of WT and homozygous KI males were examined for quantitative and qualitative parameters. Morphological assessment of spermatozoa further demonstrated that sperm structure appeared normal in 2KI mice, with no detectable abnormalities in the head, midpiece, or flagellum (Figure 2A,B). Total sperm counts did not differ significantly between KI mice and WT littermates (Figure 2C). Likewise, sperm motility, assessed under non-capacitating conditions and quantified by curvilinear velocity, showed no significant reduction in KI sperm (Figure 2D,E, Supplemental Movies S1 and S2). Consistent with these findings, additional CASA-derived kinematic parameters, including average path velocity (VAP), straight-line velocity (VSL), amplitude of lateral head displacement (ALH), beat-cross frequency (BCF), linearity (LIN), straightness (STR), and wobble (WOB), did not differ significantly between WT and KI sperm (Table 2).

### 3.3. Normal Testis and Epididymis Histology in the SPPL2A-mCherry KI Mice

**Figure 3:**
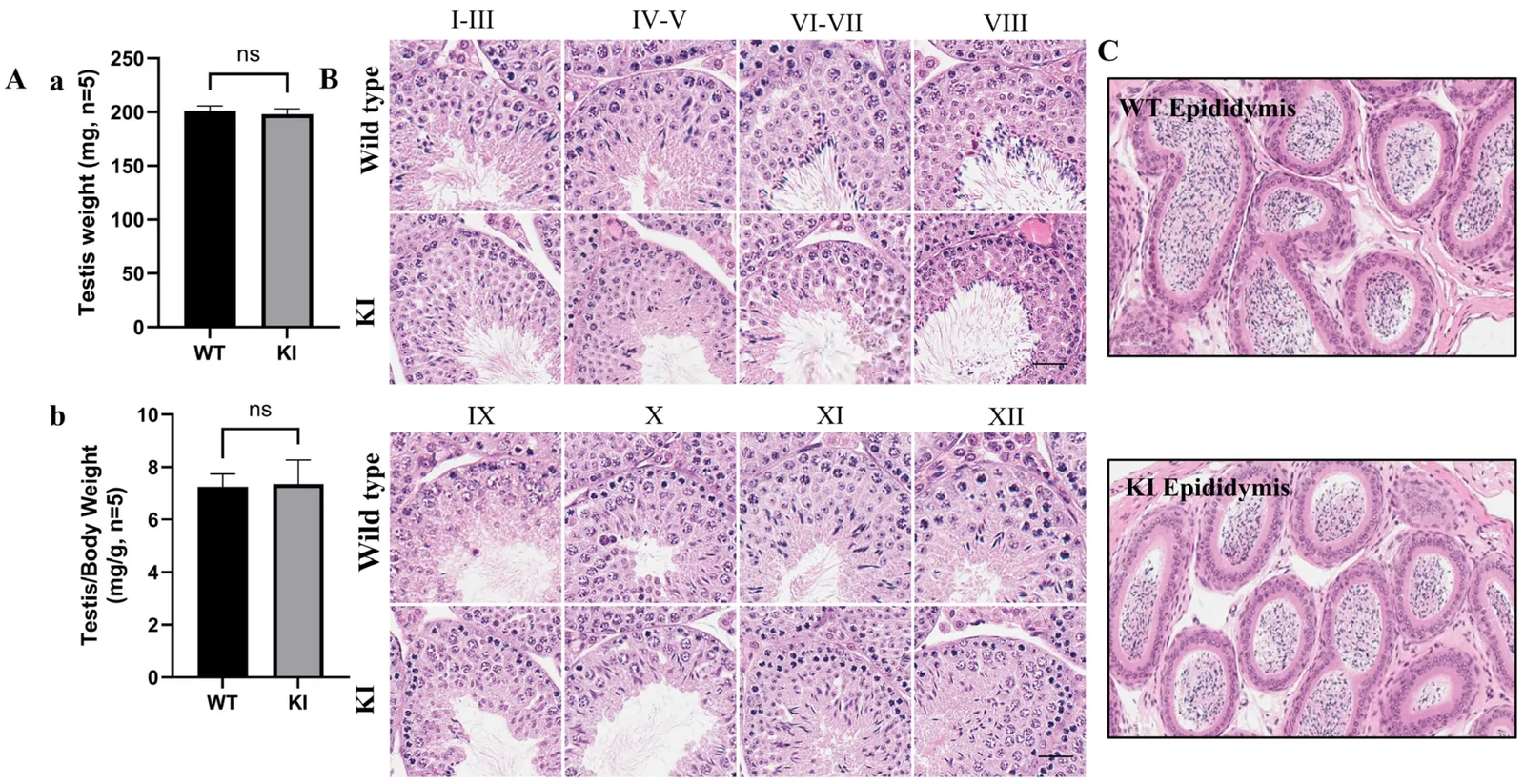
Normal spermatogenesis in the Sppl2a–mCherry KI mice. (**A**) Testis weight is unchanged in Sppl2a–mCherry KI mice. (**a**) Testis weight (mg, *n* = 5 per genotype); (**b**) Testis-to-body-weight ratio (mg/g, *n* = 5 per genotype). (**B**) Representative H&E staining of testes from WT and KI mice; (**C**) Representative H&E staining of epididymides from WT and KI mice. ns: No difference was observed between the WT mice and the KI mice.

To further investigate the spermatogenic process in the knock-in (KI) mice, a morphological examination of the testes and epididymides was carried out. Measurements revealed that both the testis weight and the ratio of testis weight to body weight in homozygous KI mice were comparable to those observed in WT controls (Figure 1D and Figure 3A). H&E staining revealed the presence of the major seminiferous epithelial stage groups in both WT and KI testes. Representative tubules from stages I–III, IV–V, VI–VII, VIII, IX, X, XI, and XII did not show obvious morphological abnormalities in KI mice compared with WT controls (Figure 3B). Moreover, the epididymal lumen in KI mice was densely populated with mature spermatozoa (Figure 3C).

### 3.4. mCherry Signal Is Detected in Testicular Cells in KI Mice

**Figure 4:**
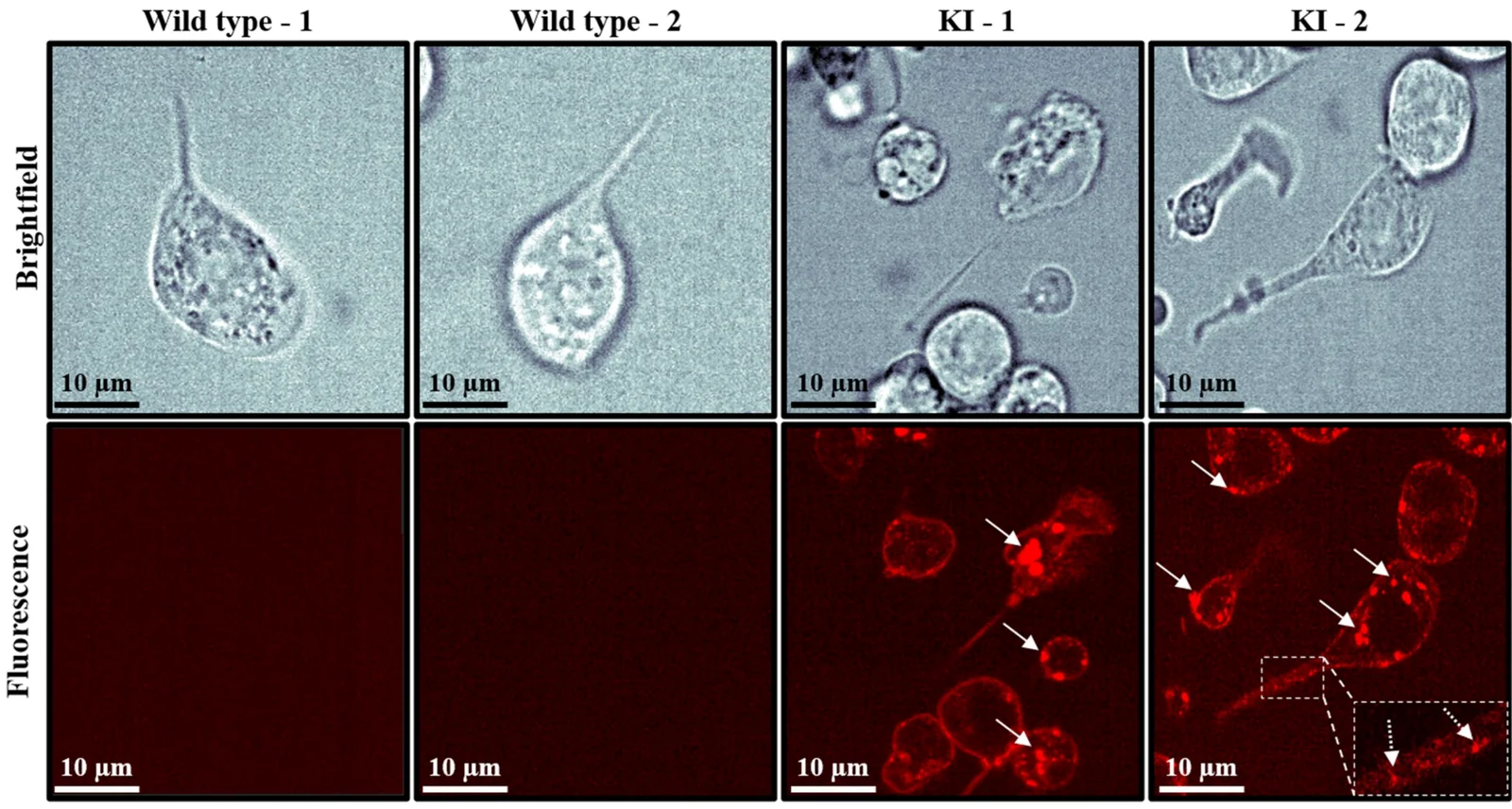
SPPL2A–mCherry fluorescence in isolated testicular germ cells from WT and KI mice. Freshly isolated living germ cells were examined by bright-field and fluorescence microscopy without fixation, permeabilization, or antibody staining. No detectable mCherry fluorescence was observed in cells from WT mice, whereas punctate SPPL2A–mCherry fluorescence was detected in germ cells from KI mice (solid arrows). Dashed arrows in the enlarged inset indicate SPPL2A–mCherry signals within the developing flagellum. Scale bars, 10 μm.

To determine whether the mCherry signal was detected in testicular cells, freshly isolated testicular cells from WT and KI mice were examined by fluorescence microscopy. No specific fluorescence signal was detected in the WT mice. In contrast, mCherry fluorescence was readily detectable in the KI mice under low magnification (Supplementary Figure S3). Intracellular mCherry signal was further examined under high magnification. mCherry signal appeared as punctate structures within the cytoplasm and was detected in elongating spermatids. Fluorescence was also observed along developing tails (Figure 4).

### 3.5. Localization of SPPL2A–mCherry During Spermatid Development

**Figure 5:**
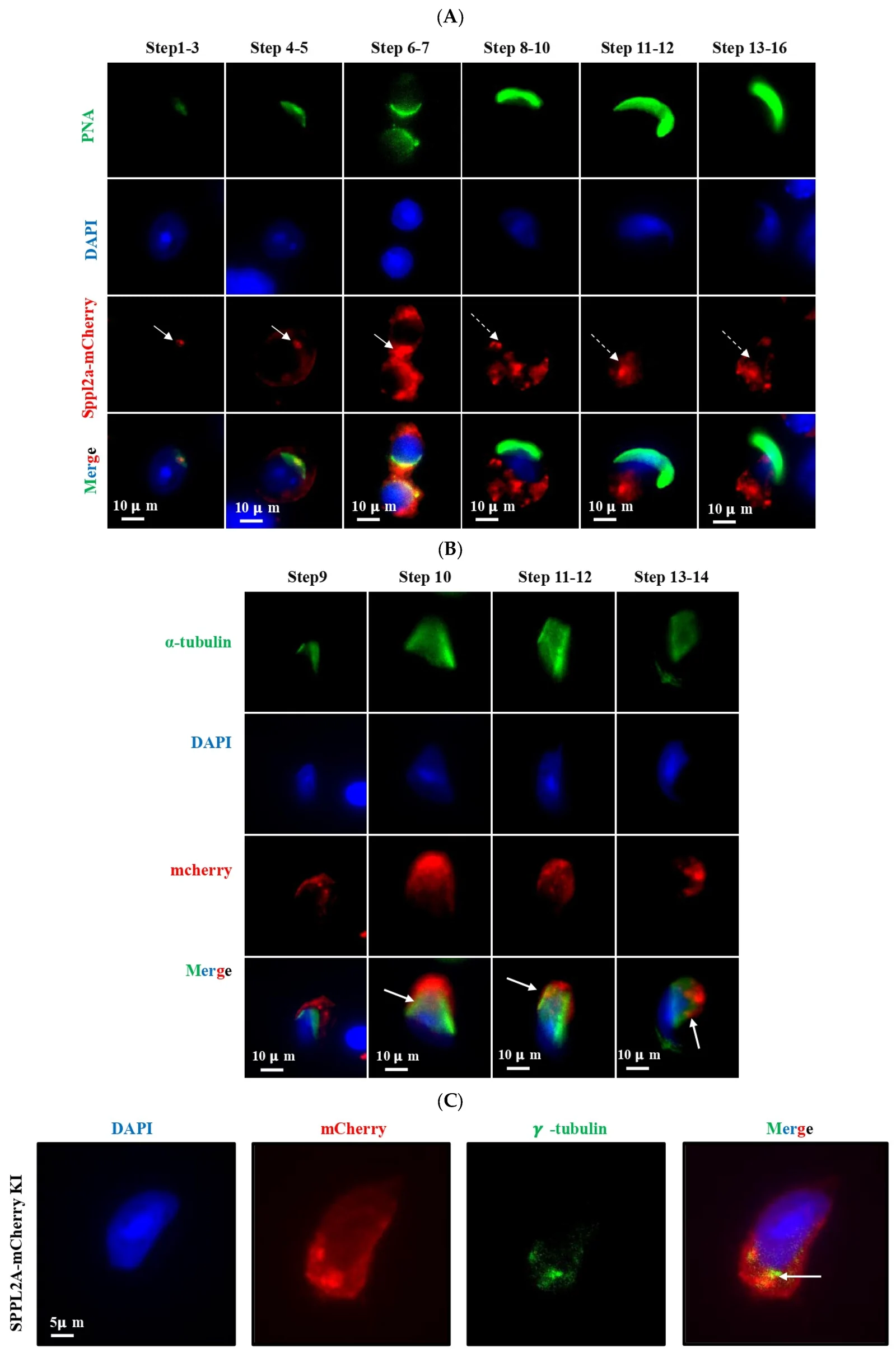
Subcellular localization of SPPL2A–mCherry during spermiogenesis. Isolated spermatids were fixed, permeabilized, and immunostained with an anti-mCherry antibody to detect SPPL2A–mCherry (red). (**A**) Spermatids at steps 1–16 were additionally stained with PNA (green) to visualize the developing acrosome and counterstained with DAPI (blue). SPPL2A–mCherry signals partially overlapped with the PNA-labeled acrosomal region during early spermiogenesis (solid arrows) and subsequently shifted toward the caudal region of the nucleus at later stages (dashed arrows). (**B**) Spermatids at steps 9–14 were additionally immunostained with an anti-α-tubulin antibody (green) to visualize the manchette and counterstained with DAPI (blue). SPPL2A–mCherry signals were detected in association with the manchette (arrows). (**C**) An elongating spermatid at approximately steps 9–10 was additionally immunostained with an anti-γ-tubulin antibody (green) and counterstained with DAPI (blue). SPPL2A–mCherry signals were detected adjacent to and partially overlapping with the γ-tubulin signal at the caudal pole of the nucleus, consistent with localization near the centrosomal region. Scale bars, 10 μm in (**A**,**B**) and 5 μm in (**C**).

To characterize the developmental distribution of SPPL2A during spermiogenesis, isolated spermatids representing different maturation stages were analyzed by fluorescence microscopy. SPPL2A-mCherry fluorescence was detectable from early round spermatids (steps 1–3) and persisted throughout subsequent stages of spermatid differentiation (Figure 5). In early spermatids, SPPL2A-mCherry appeared as discrete puncta adjacent to the nucleus. As spermiogenesis progressed, the fluorescence became increasingly concentrated within the cytoplasmic compartment surrounding the elongating nucleus and remained detectable through the final stages of spermatid maturation, indicating sustained expression throughout spermiogenesis.

To further define the subcellular localization of SPPL2A during spermatid elongation, isolated elongating spermatids were co-stained with PNA (Figure 5A), α-tubulin (Figure 5B), and γ-tubulin (Figure 5C), markers of the acrosome, manchette, and centrioles, respectively. SPPL2A–mCherry fluorescence was observed in the acrosome in early spermatids, as indicated by co-localization with PNA (Figure 5A). With the development of spermatids, the signal seemed to migrate to the cauda region of the cells. Some signals were co-localized with α-tubulin, indicating a manchette localization (Figure 5B). Some signals were present as dots in the opposite region of the acrosome. Double staining with an anti-α-tubulin antibody showed co-localization of ץ-tubulin and SPPL2A-mCherry, indicating that SPPL2A is present in the centrioles (Figure 5C).

### 3.6. SPPL2A-mCherry Trafficking in the Developing Sperm Tails

**Figure 6:**
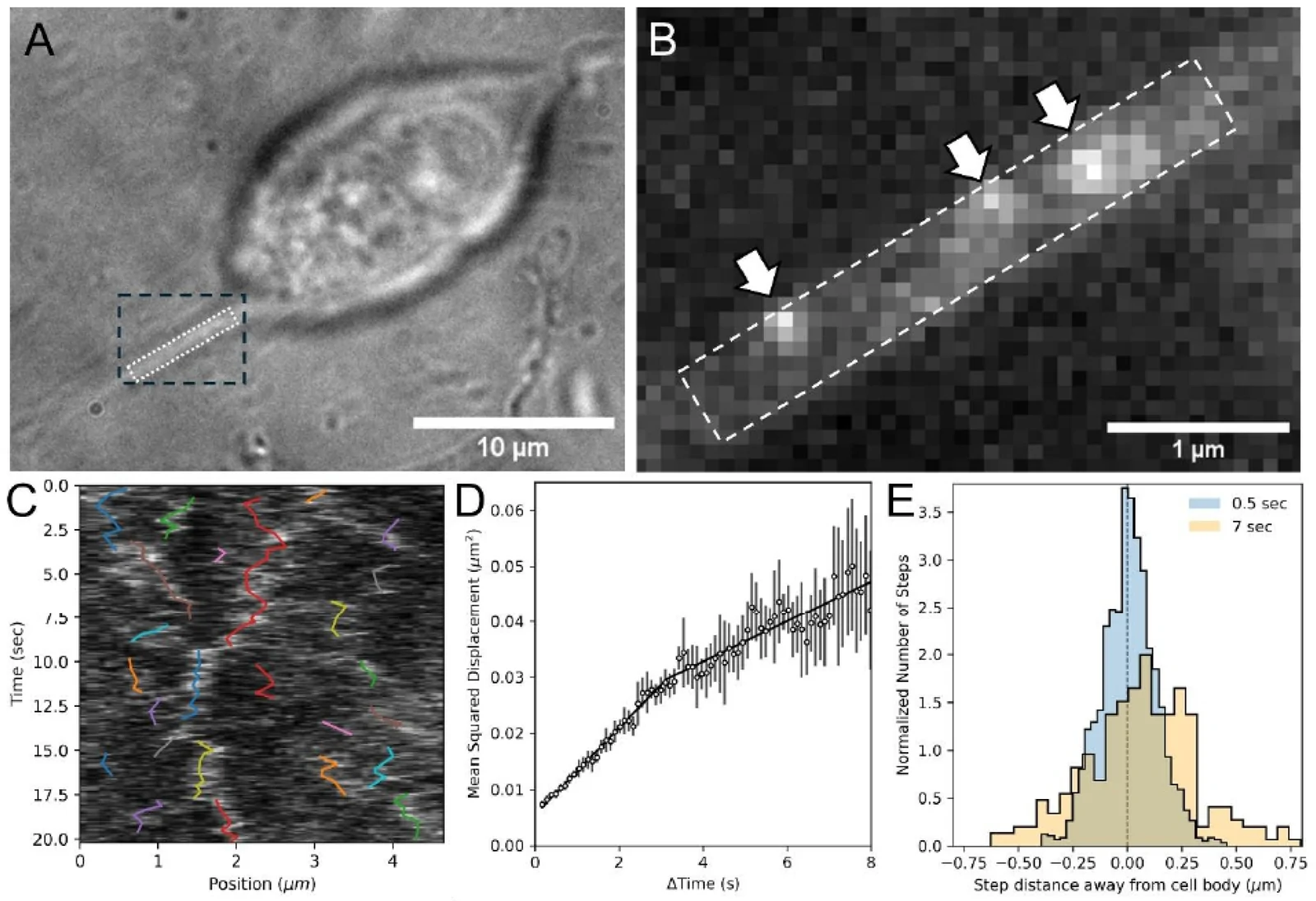
SPPL2A–mCherry preferentially moves away from the cell body in developing sperm. (**A**). Brightfield images show the sperm cell bodies and developing tails (*N* = 5 cells). The tails (e.g., black dashed box) were examined with epifluorescence imaging (**B**) to track the dynamics of SPPL2-GFP puncta (white arrows, Supplemental Movies S3 and S4). (**C**) Kymographs of intensity versus time and distance along the tails revealed diffusion and trafficking of SPPL2a. Individual SPPL2a vesicles were tracked (colored lines) and analyzed. (**D**) Mean-squared displacement analysis demonstrated two diffusion regimes and a confinement length scale of 153 ± 15 nm, consistent with hopping diffusion and a confinement time of 3.1 ± 0.5 s. (**E**) At both the short times (Δt = 0.5 s, *p* = 0.03) and long times (Δt = 7 s, *p* = 0.0002), SPPL2A-mCherry preferentially trafficked away from the cell body; significance was assessed using a one-sample *t*-test to determine that the mean step was away from the cell body.

The diffusion and drift analysis of SPPL showed that SPPL2 primarily behaved like a random, confined diffuser within the developing sperm tail. However, a slight and statistically significant bias in the SPPL2 motion occurred away from the cell body. A non-linear least-squares fitting of the MSD vs. Δt to a two-regime diffusion model, as shown in Equation (1), yielded. $D_{short}$ = 0.0038 ± 0.0004 µm^2^/s, $D_{long}\;$= 0.0018 ± 0.0002 µm^2^/s, $\tau_{c}$ = 3.1 ± 0.5 s, and σ = 74 ± 12 nm. This diffusion rate is significantly slower than expected for a small, free vesicle or protein cluster, and is consistent with highly confined or restricted motion. Additionally, the transition between $D_{short}$ and $D_{long}$ a confinement length scale of 153 ± 15 nm indicates a sub-diffraction-limited barrier to SPPL2 motion within the tail (Figure 6).

Analysis of the average step direction revealed a subtle but significant motion of SPPL2 away from the cell body. At short time intervals (Δt = 0.5 s), the displacement distribution shifted toward positive values, indicating preferential movement toward the distal region of the developing tail (*p* = 0.03). This directional bias became more pronounced at longer time intervals (Δt = 7 s), with particles exhibiting significantly greater displacement away from the cell body (*p* = 0.0002). Representative time-lapse imaging of SPPL2A–mCherry particle movement is shown in the accompanying video, with additional examples provided (Supplementary Movies S3 and S4).

## 4. Discussion

In the present study, we generated a SPPL2A-mCherry knock-in mouse model to investigate the localization and trafficking behavior of SPPL2A during spermatogenesis. Homozygous knock-in mice displayed normal spermatogenesis, sperm morphology, and fertility, indicating that the mCherry tag did not disrupt the physiological function of SPPL2A. Using live-cell imaging and single-particle tracking, we observed that SPPL2A forms punctate structures within developing sperm flagella and undergoes dynamic trafficking along the elongating tail. Quantitative trajectory analysis further revealed that SPPL2A exhibits restricted diffusion with a defined confinement length scale, as well as a slight directional bias away from the cell body. These observations indicate that SPPL2A-containing puncta are not entirely static within the developing sperm flagellum and are consistent with intracellular transport during spermiogenesis. Since the formation of sperm flagella requires coordinated delivery of structural and regulatory proteins to the elongating axoneme, the dynamic trafficking behavior of SPPL2A suggests that it may play a role in the intracellular transport pathways involved in flagellar assembly. Motile cilia and flagella are highly specialized microtubule-based organelles that require precise spatial and temporal coordination of protein transport for their assembly and maintenance [1,4].

The assembly of cilia and flagella is critically dependent on IFT, a conserved bidirectional transport system that delivers cargo proteins along axonemal microtubules [4,5,8]. IFT is mediated by two large protein complexes, IFT-A and IFT-B, which cooperate with kinesin-2 and dynein-2 motors to transport cargo toward the distal tip and back toward the basal body, respectively [10]. In the context of spermatogenesis, several studies have demonstrated that disruption of IFT components leads to severe defects in sperm flagellar assembly and male infertility. For example, genetic ablation of IFT25, IFT27, or IFT172 results in abnormal axoneme formation and defective spermiogenesis in mice [8,9]. Notably, IFT27 functions as a small Rab-like GTPase that regulates the loading and turnover of membrane-associated cargo within the IFT complex [11]. In our previous yeast two-hybrid screening, SPPL2A was identified as a potential binding partner of IFT27, suggesting a possible link between SPPL2A and IFT-mediated trafficking. The localization and movement of SPPL2A-containing puncta observed in the present study are therefore consistent with the possibility that SPPL2A may be associated with membrane-related transport during sperm flagellar assembly.

To investigate the localization and trafficking behavior of endogenous SPPL2A during spermatogenesis, we generated a SPPL2A–mCherry knock-in mouse model. Traditional approaches for studying protein localization in germ cells often rely on antibody-based immunostaining or small epitope tags such as HA, FLAG, or V5. While these strategies are valuable for determining static localization patterns, they do not permit direct visualization of protein dynamics in living cells [16]. In contrast, fluorescent protein knock-in models enable real-time monitoring of endogenous proteins under physiological expression levels and have become powerful tools for studying intracellular trafficking processes [17,18,19]. Fluorescently tagged mouse models have been widely used in cilia and flagella research, including ARL13B–mCherry, Centrin2–GFP, and several IFT reporter lines, providing important insights into ciliary assembly and protein transport [20]. Because sperm flagellar formation is a highly dynamic process involving continuous cargo movement along microtubule-based structures, direct visualization of endogenous proteins represents a significant advantage over conventional fixed-cell approaches.

Notably, homozygous SPPL2A–mCherry mice displayed normal spermatogenesis, sperm morphology, and fertility, indicating that fusion of mCherry to SPPL2A did not substantially impair its physiological function. This observation is particularly relevant because fluorescent proteins are considerably larger than conventional epitope tags and may potentially interfere with protein folding, trafficking, or enzymatic activity. The normal reproductive phenotype, therefore, supports the use of this knock-in model for studying endogenous SPPL2A behavior during spermatogenesis. Examination of isolated germ cells revealed that SPPL2A–mCherry was organized into discrete punctate structures rather than displaying diffuse cytoplasmic localization. The punctate distribution of SPPL2A is noteworthy because proteins undergoing active intracellular transport are frequently assembled into cargo-containing particles rather than remaining freely dispersed within the cytoplasm. Similar punctate structures have been described for multiple components of the IFT machinery and for cargo proteins undergoing microtubule-dependent trafficking during spermatid differentiation. Furthermore, SPPL2A became progressively enriched during the elongation phase of spermiogenesis, coinciding with the onset of manchette formation and rapid flagellar assembly [21,22]. This temporal relationship indicates that recruitment of SPPL2A is developmentally regulated and may be linked to the increased demand for protein transport that accompanies sperm tail biogenesis. Importantly, co-localization with α-tubulin demonstrated a gradual redistribution of SPPL2A from the perinuclear region toward microtubule-rich manchette and flagellar structures. Because the manchette functions as a major transport platform that mediates intramanchette transport (IMT) and coordinates delivery of proteins to the developing sperm tail [2,21,22], the observed spatial transition of SPPL2A is consistent with its incorporation into microtubule-dependent trafficking pathways.

Consistent with this interpretation, Single-particle tracking analysis revealed that SPPL2A exhibits slow diffusion and confined motion within the developing sperm tail. The measured diffusion coefficients were considerably lower than those expected for freely diffusing cytosolic proteins. In addition, mean-squared displacement analysis identified a confinement length scale of 153 ± 15 nm, indicating that SPPL2A movement is spatially restricted within nanoscale domains of the flagellum. Such confined diffusion behavior is commonly observed for proteins associated with membrane microdomains or multiprotein complexes, rather than freely diffusing molecules [23]. In ciliary membranes, many signaling and transport proteins display restricted motion due to interactions with underlying axonemal structures or transport machinery such as IFT particles [2,5]. Therefore, the diffusion properties observed for SPPL2A are consistent with a model in which the protein associates with membrane-associated transport complexes within the developing sperm flagellum.

SPPL2A-containing particles exhibited directional displacement along the developing flagellum; trajectory analysis revealed a subtle directional bias away from the cell body. In motile cilia and flagella, anterograde transport from the basal body to the distal tip is primarily driven by kinesin-2 motors, whereas retrograde transport is mediated by dynein-2 motors [24,25]. The slight directional bias observed in our analysis suggests that SPPL2A particle movement may not be entirely random; however, the underlying transport machinery remains unknown. During spermiogenesis, continuous delivery of proteins to the distal tip is required to support the rapid extension of the flagellum and the assembly of axonemal and accessory structures [2,26]. The observed particle behavior may reflect the movement of SPPL2A-containing membrane-associated structures within the growing flagellum [24,27].

SPPL2A is a member of the signal peptide peptidase-like (SPPL) family of intramembrane aspartyl proteases, which regulate diverse cellular processes through cleavage of type II transmembrane proteins [13,28]. Beyond its established proteolytic activity, accumulating evidence indicates that SPPL2A is closely associated with membrane protein trafficking and turnover through regulation of endosomal transport pathways [28,29]. The dynamic localization and transport behavior observed in the present study therefore suggest that SPPL2A may serve functions beyond proteolytic processing during spermiogenesis. Given its localization within developing sperm flagella and its apparent association with transport-related structures, SPPL2A may contribute to the trafficking, maturation, or turnover of membrane-associated proteins required for flagellar assembly. Although the molecular substrates of SPPL2A in germ cells remain unknown, our findings raise the possibility that intramembrane proteolysis and protein transport may be functionally coordinated during sperm tail formation.

**Figure 7:**
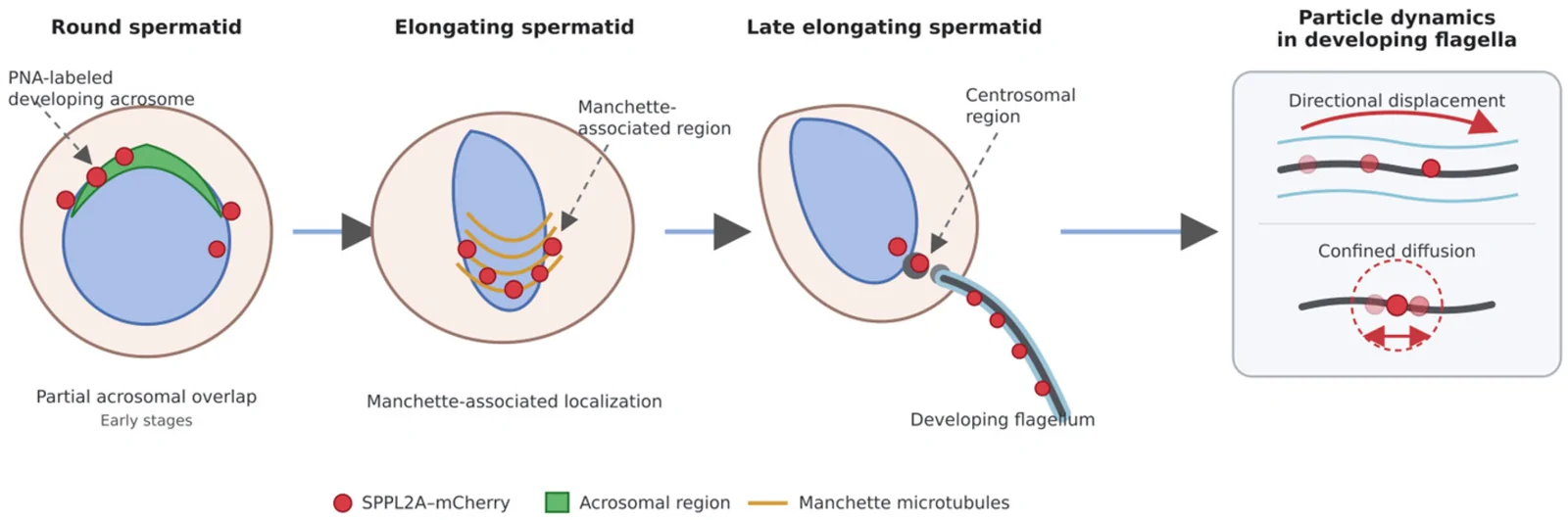
Schematic summary of the stage-dependent localization and particle behavior of SPPL2A during mouse spermiogenesis. SPPL2A–mCherry signals partially overlapped with the PNA-labeled developing acrosomal region during early spermiogenesis and were subsequently detected in association with the manchette, near the centrosomal region, and within the developing flagellum during spermatid elongation. Single-particle tracking revealed directional displacement and confined diffusion of SPPL2A-containing particles within developing flagella. Faded red circles indicate successive positions of the same particle, whereas the short double-headed arrow represents local movement within a confined domain rather than long-range bidirectional transport. Arrows between developmental stages indicate the temporal sequence of the observed localization patterns and do not demonstrate direct transport of SPPL2A between these subcellular regions.

Taken together, our findings reveal a stage-dependent redistribution of SPPL2A during mouse spermiogenesis. As summarized in Figure 7, SPPL2A–mCherry signals were initially detected in partial overlap with the developing acrosomal region, followed by association with the manchette and subsequent localization near the centrosomal region and within the developing flagellum. Single-particle tracking further identified directional displacement and confined diffusion of SPPL2A-containing particles in developing flagella. These observations suggest that SPPL2A undergoes spatially and temporally regulated trafficking during spermatid differentiation. Nevertheless, although SPPL2A was initially identified as a candidate IFT27-interacting protein through yeast two-hybrid screening, the present findings do not establish SPPL2A as an IFT cargo or demonstrate that its trafficking is mediated by IFT27. Further biochemical and functional studies are required to define the relationship between SPPL2A and the IFT machinery.

## 5. Conclusions

In conclusion, SPPL2A exhibits stage-dependent redistribution and distinct particle behaviors during mouse spermiogenesis. The SPPL2A–mCherry KI mouse provides a useful model for investigating the localization and trafficking of SPPL2A in developing germ cells. Further biochemical and functional studies are required to determine whether IFT27 or other components of the IFT machinery contribute to SPPL2A trafficking during flagellar development.

## Figures and Tables

**Table 1:** Fertility and fecundity of the WT and Sppl2a-mCherry KI mice.

Genotype	Fertility	Litter Size
WT	4/4	8.25 ± 1.26
Sppl2a-mCherry KI (Ho)	4/4	8.00 ± 0.82

**Table 2:** Additional CASA-derived sperm kinematic parameters in wild-type and Sppl2a-mCherry KI mice.

Parameter	WT (*n* = 5)	KI (*n* = 5)	*p* Value
VAP (μm/s)	123.58 ± 0.51	121.32 ± 7.34	0.767
VSL (μm/s)	71.35 ± 1.86	69.54 ± 4.28	0.708
ALH (μm)	16.32 ± 0.04	16.15 ± 0.47	0.728
BCF (Hz)	27.36 ± 0.99	26.58 ± 1.57	0.685
LIN (%)	28.64 ± 0.58	27.36 ± 0.82	0.238
STR (%)	57.52 ± 2.65	54.22 ± 0.72	0.264
WOB (%)	47.69 ± 0.43	48.31 ± 2.65	0.823

Note: Data are presented as mean ± SEM (*n* = 5 mice per group). Statistical comparisons between WT and Sppl2a-mCherry KI mice were performed using an unpaired two-tailed Student’s *t*-test. Abbreviations: VAP, average path velocity; VSL, straight-line velocity; ALH, amplitude of lateral head displacement; BCF, beat cross frequency; LIN, linearity; STR, straightness; WOB, wobble.

## Data Availability

The original contributions presented in this study are included in the article/Supplementary Material. Further inquiries can be directed to the corresponding authors.

## Supplementary Materials

The following supporting information can be downloaded at: https://www.mdpi.com/article/10.3390/cells15191773/s1, Figure S1: Strategy to generate Sppl2a–mCherry KI mice; Figure S2: A representative PCR result for genotyping; Figure S3: Examination of mCherry signal in isolated testicular cells from WT and KI mice under low magnification; Movie S1: Representative CASA recording of sperm motility from a wild-type mouse; Movie S2: Representative CASA recording of sperm motility from a Sppl2a–mCherry knock-in mouse; Movie S3: SPPL2A–mCherry particle movement in a developing sperm tail (Sample 1); Movie S4: SPPL2A–mCherry particle movement in a developing sperm tail (Sample 2).
