# Supplementary material for: Localization and Trafficking Behavior of SPPL2A During Mouse Spermiogenesis
Source: Cells. 2026 Sep 29;15(19):1773. doi: 10.3390/cells15191773 (PMC13664308; doi:10.3390/cells15191773)
Supplement: Supplementary file 1 [file cells-15-01773-s001.zip › cells-4466460-supplementary-figures.pdf]

A

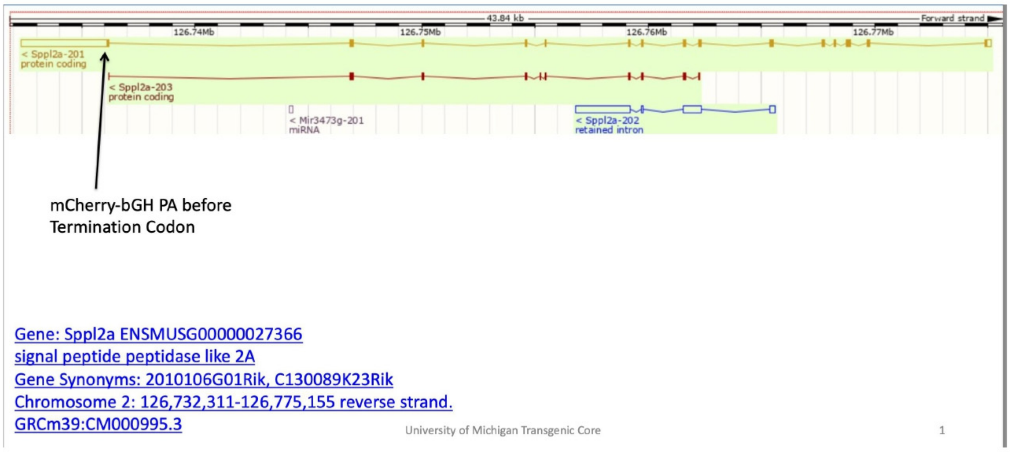
Forward strand
< Sppl2a-201
protein coding
< Sppl2a-203
protein coding
< Mir3473g-201
miRNA
< Sppl2a-202
retained intron
mCherry-bGH PA before
Termination Codon
Gene: Sppl2a ENSMUSG00000027366
signal peptide peptidase like 2A
Gene Synonyms: 2010106G01Rik, C130089K23Rik
Chromosome 2: 126,732,311-126,775,155 reverse strand.
GRCm39:CM000995.3
University of Michigan Transgenic Core
1

B

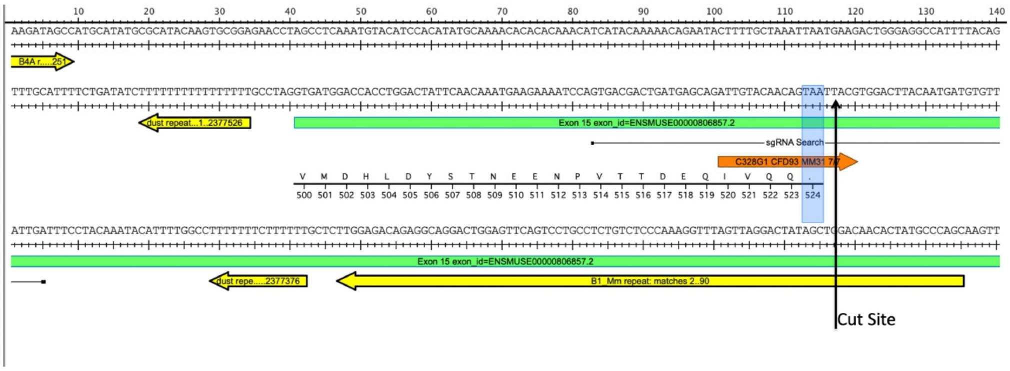
Exon 15 exon_id=ENSMUSE00000806857.2
sgRNA Search
B1_Mm repeat: matches 2..90
Cut Site

C

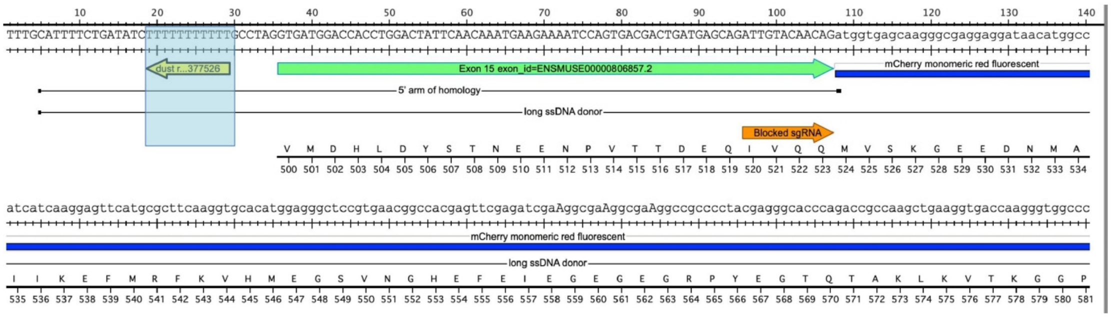
Exon 15 exon_id=ENSMUSE00000806857.2
mCherry monomeric red fluorescent
5' arm of homology
long ssDNA donor
Blocked sgRNA

D

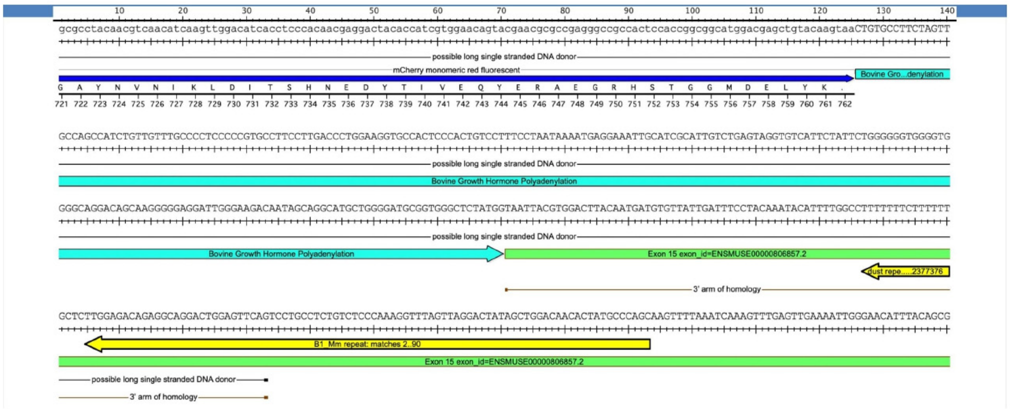

Figure S1: Strategy to generate Sppl2a–mCherry KI mice

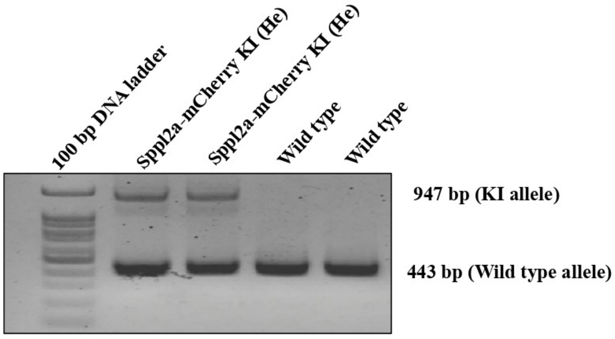

Figure S2: A representative PCR result for genotyping

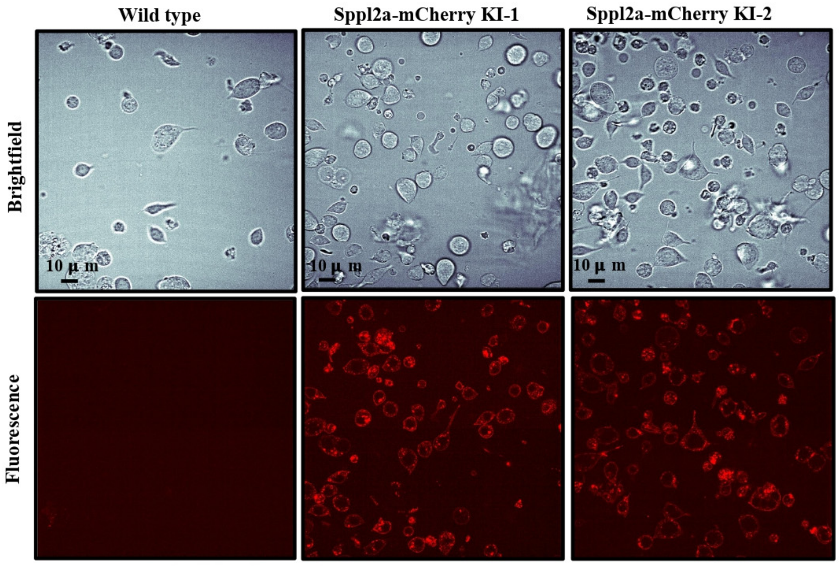

Figure S3: Examination of mCherry signal in isolated testicular cells from WT and KI mice under low magnification
